# Plasticization of Polylactide with Myrcene and Limonene as Bio-Based Plasticizers: Conventional vs. Reactive Extrusion

**DOI:** 10.3390/polym11081363

**Published:** 2019-08-18

**Authors:** Berit Brüster, Yann-Olivier Adjoua, Reiner Dieden, Patrick Grysan, Carlos Eloy Federico, Vincent Berthé, Frédéric Addiego

**Affiliations:** Luxembourg Institute of Science and Technology (LIST), Department Materials Research and Technology (MRT), ZAE Robert Steichen, 5 Rue Bommel, L-4940 Hautcharage, Luxembourg

**Keywords:** polylactide, plasticization, myrcene, limonene, reactive extrusion, structure, mechanical properties

## Abstract

Polylactide (PLA) was blended by conventional and reactive extrusion with limonene (LM) or myrcene (My) as bio-based plasticizers. As-processed blends were carefully analyzed by a multiscale and multidisciplinary approach to tentatively determine their chemical structure, microstructure, thermal properties, tensile and impact behaviors, and hydrothermal stability. The main results indicated that LM and My were efficient plasticizers for PLA, since compared to neat PLA, the glass transition temperature was reduced, the ultimate tensile strain was increased, and the impact strength was increased, independently of the type of extrusion. The addition of a free radical initiator during the extrusion of PLA/LM was beneficial for the mechanical properties. Indeed, the probable formation of local branched/crosslinked regions in the PLA matrix enhanced the matrix crystallinity, the tensile yield stress, and the tensile ultimate stress compared to the non-reactive blend PLA/LM, while the other properties were retained. For PLA/My blends, reactive extrusion was detrimental for the mechanical properties since My polymerization was accelerated resulting in a drop of the tensile ultimate strain and impact strength, and an increase of the glass transition temperature. Indeed, large inclusions of polymerized My were formed, decreasing the available content of My for the plasticization and enhancing cavitation from inclusion-matrix debonding.

## 1. Introduction

Polylactide (PLA) is a highly versatile aliphatic, compostable polymer derived from 100% annually renewable resources. PLA is reported to be a viable alternative to petrochemical-based plastics for many applications [1]. One of the drawbacks of PLA is its low chain mobility due to both high intrinsic chain rigidity and low entanglement density, engendering a low molecular deformability when a strain is applied [2]. This results in a high elastic modulus, low tensile elongation at break, and low toughness, limiting the engineering application field of PLA in its unmodified state. To address this issue, chain mobility is enhanced by mixing PLA with a plasticizer by extrusion, decreasing the glass transition temperature $T_{g}$ and increasing molecular deformability [3,4]. The final mechanical performance of the materials depends on numerous parameters including the plasticizer weight fraction and molecular weight [5]. In general, to avoid initial phase separation between the PLA matrix and the plasticizer, a weight fraction of maximum 20 wt% is suitable concerning the plasticizer. Low molecular weight plasticizers provide a high depression of PLA $T_{g}$ (for example Tributyl citrate (TbC) with a $M_{w}$ of 360 g·mol^−1^ decreases the $T_{g}$ of PLA from 52 °C to 25 °C), but are known to progressively engender phase separation followed by leaching of the plasticizer [3]. Obviously, such an ageing mechanism is not desirable for a long-term application [3,6]. On the contrary, selecting a high molecular weight plasticizer provides a lower decrease of $T_{g}$ (for example oligoester diethyl bishydroxymethyl malonate (DBMAT) with a $M_{w}$ of 3200 g·mol^−1^ decreases the $T_{g}$ of PLA from 52 °C to 39 °C) and a higher phase stability [3]. As highlighted in Reference [5], low molecular weight plasticizers can be partially grafted onto PLA backbone by reactive extrusion [7], to avoid phase separation. In particular, during this extrusion procedure, the plasticizer and PLA are blended in the presence of a free radical initiator resulting in a grafting of around 50% of the initial plasticizer (based on Soxhlet extraction testing) and intermediate mechanical properties between those of unmodified PLA and those of PLA plasticized by conventional extrusion [8,9]. In a recent paper, it has been found that plasticization by reactive extrusion induces the formation of plasticizer inclusions with a heterogeneous structure, the shell being rich in polymerized plasticizer and the core being less rich in polymerized plasticizer [10]. This finding further highlights the structural complexity of PLA plasticized by reactive extrusion. In this context, poly(ethylene glycol) (PEG, $M_{w}$ ranging from 380 g·mol^−1^ to 550 g·mol^−1^) [5,11], and poly(ethylene glycol) methyl acrylate (acryl-PEG, $M_{w}$ ranging from 480 g·mol^−1^ to 550 g·mol^−1^) [8,9,10,12,13] have been successfully used. Unfortunately, neither of these two plasticizers is bio-based, which would have been desirable in order to retain the sustainability of PLA.

Among all available natural substances that can be used as plasticizer, terpenes, a family of unsaturated reactive molecules that can be extracted from by-products, would be suitable. Limonene (LM) and myrcene (My) are among the most abundant representatives of this family. In particular, LM is the most abundant monocyclic monoterpene and is the main component in the oil of citrus fruit peel [14,15]. LM with a $M_{w}$ of about 136 g·mol^−1^ has been proved to be miscible with PLA, and hence, has been successfully used to plasticize PLA by conventional extrusion in a pioneer work leading to a transparent film up to a LM fraction of 20 wt% [16]. A significant decrease of $T_{g}$ from 60.3 °C for unmodified PLA to 33.8 °C for PLA/LM 80/20 (in wt%/wt%) is obtained, resulting at the same time in a decrease of the elastic modulus from 1350 MPa to 770 MPa, and an increase of elongation at break from 1.5% to 165% [16]. No impact testing has however been performed in this previous study to verify if the plasticizing effect was also active concerning toughness. Furthermore, to our best knowledge, no attempt has been made to graft LM onto PLA backbone by reactive extrusion. Concerning My, it is the most abundant terpene in cannabis reaching at maximum 50 vol% of the overall terpene content in a cannabis plant. My is a highly reactive monomer that can undergo spontaneous radical thermal polymerization [17,18], and hence, is generally provided mixed with an inhibitor and has to be stored in a refrigerator. To our best knowledge, no paper has been found relating the use of My to plasticize PLA. Furthermore, My could present the ability to polymerize when submitted to heat as in the case of an extrusion procedure, conducting to a new material with an original structure, and hence, to potentially novel mechanical performance. In the presence of peroxide, a potential grafting of My onto PLA backbone can also be possible during reactive extrusion since, as LM, My monomers possess double bonds. 

In this work, we hence propose to plasticize PLA with LM and My by conventional and reactive extrusion, and to analyze the resulting material structure and properties. During the reactive extrusion, three main reactions are expected: (i) the free radical initiated branching and crosslinking of PLA, (ii) the grafting/crosslinking of the plasticizers onto PLA backbone, and (iii) the polymerization of the plasticizers. The occurrence of these reactions has been studied by a multiscale and multidisciplinary approach. The processability between PLA and the plasticizers is first analyzed by monitoring the evolution of extrusion force as a function of time and by measuring the material molecular weight by size exclusion chromatography (SEC). The chemical structure of the materials is investigated by proton nuclear magnetic resonance (^1^H NMR), while their microstructure is characterized by micro-computed x-ray tomography (µCT) and atomic force microscopy (AFM). Thermal properties are measured by differential scanning calorimetry (DSC), while mechanical properties are evaluated by tensile and impact testing. Finally, the stability of plasticized PLA blends is investigated by hydrothermal ageing.

## 2. Materials and Methods

### 2.1. Materials and Solubility

PLA was supplied by NatureWorks (Minnetonka, MN, USA) under the reference 4043D and contained 4.2% of d-lactide units. The density of PLA was 1.24 g·cm^−3^ based on the supplier datasheet. (R)-(+)-Limonene ((R)-4-Isopropenyl-1-methyl-1-cyclohexene with acronym LM) was purchased from Sigma-Aldrich (Steinheim, Germany). LM was characterized by a molecular weight $M_{w}$ of about 136.2 g·mol^−1^ and a density at 20 °C of 0.842 g·mL^−1^. β-Myrcene (7-Methyl-3-methylene-1,6-octadiene with acronym My) was obtained from Sigma-Aldrich (Steinheim, Germany). My was characterized by a molecular weight $M_{w}$ of about 136.2 g·mol^−1^ and a density of 0.791 g·mL^−1^. It is also to be noted that My contained butylated hydroxytoluene (BHT) as inhibitor (1000 ppm). The organic peroxide 2,5-bis(*tert*-butylperoxy)-2,5-dimethylhexane was used as a free radical initiator for the reactive extrusion. It was purchased from Sigma-Aldrich (Steinheim, Germany) with the reference Luperox 101 (L101) and exhibited a half-lifetime of 1 min at 180 °C. Since extrusion procedures were conducted at 180 °C for several minutes, it was highly probable that L101 totally decomposed during the time required for the reactive plasticization, which appeared desirable. Furthermore, it is important to note that L101 was also recognized by the U.S. Food and Drug Administration (FDA) as an additive (accelerator) for rubber packaging material compatible with food under certain conditions (fraction of maximum 1.5 wt% in a rubber matrix) [19].

To start with, it was of interest to quantify the solubility between PLA and the two bio-based plasticizers. To this end, the Hansen Solubility Parameters (HSP) were determined for PLA, LM, and My by means of the software Hansen Solubility Parameters in Practice (HSPiP, Steven Abbott and Hiroshi Yamamoto, version 5.0.13 x64) [20]. In HSP theory, the solubility δ of a substance was split into three terms, which represented the different structural contributions from dispersion forces $\delta_{d}$, from polar forces $\delta_{p}$, and from hydrogen bonding $\delta_{h}$, as follows [21,22]:

(1) $$\delta =\;{\left(\delta_{d}^{2}{+\;\delta }_{p}^{2}{+\delta }_{h}^{2}\right)}^{0.5}$$

Each contribution was expressed based on the following equations:

(2) $$\delta_{d}=\;\frac{\sum F_{\mathrm{di}}}{V_{m}}$$

(3) $$\delta_{p}=\;\frac{{\left(\sum F_{\mathrm{pi}}^{2}\right)}^{0.5}}{V_{m}}$$

(4) $$\delta_{h}=\;{\left(\frac{\sum E_{\mathrm{hi}}}{V_{m}}\right)}^{0.5}$$

where each structural group’s contribution to the different interactions was represented by $F_{\mathrm{di}}$ (dispersion forces), $F_{\mathrm{pi}}$ (polar forces), and $E_{\mathrm{hi}}$ (hydrogen bonding), while $V_{m}$ was the molar volume of the substance. The strength of this theory relied on its three-dimensional interpretation. In fact, each of the three contribution parameters was considered as a coordinate in space. Since the plasticizer and the polymer had both some specific contribution parameters, it was therefore possible to calculate the spatial distance between them, and so to determine their thermodynamic similarity degree. Thereby, the acceptance degree of the polymer was defined as the radius of a sphere. The plasticizer whose coordinates were located inside the sphere was expected to dissolve or swell the polymer. On the contrary, the plasticizer those coordinates were outside the sphere was not miscible with the polymer. Finally, the closer both plasticizer and polymer coordinates were, the better the compatibility. Based on HSP theory, the distance $R_{a}$ between PLA and a plasticizer (Plast) was determined by:

(5) $$R_{a}=\;{\left[4{\left(\delta_{d\;\mathrm{Plast}}\;-\;\delta_{d\;\mathrm{PLA}}\right)}^{2}+{\left(\delta_{p\;\mathrm{Plast}}\;-\;\delta_{p\;\mathrm{PLA}}\right)}^{2}+{\left(\delta_{h\;\mathrm{Plast}}\;-\;\delta_{h\;\mathrm{PLA}}\right)}^{2}\right]}^{0.5}$$

Subsequently, the relative energy difference, named the RED number, was calculated based on:

(6) $$\mathrm{RED}=\;\frac{R_{a}}{R_{o}}$$

where $R_{o}$ corresponded to the PLA sphere radius taken equal to 10.7 (MJ·m^−3^)^0.5^ [23]. Therefore, the closer the RED value was to zero, the better the compatibility between PLA and the plasticizer was. An RED value higher than 1 indicated no miscibility between the plasticizer and the matrix. The HSP parameters of PLA, LM, and My determined with HSPiP software, and the corresponding RED numbers were listed in Table 1. As a comparison, the same calculations were done for PEG, known to be a good plasticizer for PLA [5,11]. It can be noted that the RED number of LM was 0.93 and that of My was 0.99, showing a borderline solubility with PLA [23], while PEG exhibited a higher solubility with PLA based on a RED value of 0.29. Nevertheless, LM appeared slightly more miscible with PLA compared to My. The calculated contributions of dispersion forces $\delta_{d}$ and polar forces $\delta_{p}$ of PLA, LM and My were quite similar to the ones determined experimentally, but the calculated contributions of the hydrogen bonding $\delta_{h}$ were systematically lower than the experimental ones [24,25]. Based on HSP results, a possible phase separation may be expected between PLA and LM or My during the processing, forming inclusions as already observed for example in the case of acryl-PEG plasticizer [8,9,10].

### 2.2. Processing

All the PLA blends were extruded with a 15 cc twin-screw DSM Xplore micro-compounder (Geleen, The Netherlands), by means of nitrogen as purging gas. The barrel temperature was set to a constant temperature of 180 °C for all the formulations, corresponding to a melt temperature ranging between 170 °C and 175 °C. Screw speed was set to 50 rpm, while a residence time of 5 min was selected. One batch of 6 g was introduced into the micro-compounder for each extrusion procedure, while several batches were processed per formulation. Note that lower screw speeds and higher screw speeds were tested but provided lower mechanical properties when compared to 50 rpm, explaining why this speed was chosen for the actual works. Before processing, PLA pellets were dried for at least 12 h at 60 °C in an oven Thermo Scientific Heraeus (Langenselbold, Germany) under reduced pressure. During the experiments, the liquids (LM, My, and L101) were kept in a closed dewar containing liquid nitrogen to prevent any potential photo-polymerization and any degradation of the plasticizers by direct contact with the ambient humidity. PLA was first introduced and melted in the extruder. LM (or My) and L101 were mixed in a small glass vial, and then injected into the extruder using a syringe. The evolution of the force (in N) during the extrusion was systematically recorded. The following formulations of PLA/(LM or My)/L101 were processed: 100/0/0, 80/20/0 and 79/20/1 (in wt%/wt%/wt%). The materials were extruded into strands of diameter 3-5 mm and then were ground into flakes of size 1-3 mm by means of a micro-grinder Wanner Baby B08.10 (Wertheim, Germany). Tensile and impact specimen were prepared by means of an injection molding machine HAAKE MiniJet II (Karlsruhe, Germany) from the flakes. Injection procedures were conducted at 180 °C, setting the mold temperature to 65 °C. A pressure of 600 bars was used for the injection during 5 s and then a post-pressure of 300 bars was used during 5 s to compensate for sample shrinkage. The dimensions of the impact specimens were 60 mm × 11 mm × 3 mm, while tensile specimen dimensions complied with the standard ASTM D638 type V.

### 2.3. Characterization

#### 2.3.1. Size Exclusion Chromatography

The weight average molecular weight $M_{w}$ and the number average molecular weight $M_{n}$ of the extruded and injected materials (neat PLA and PLA blends) were determined by size exclusion chromatography (SEC). This analysis was also done in the case of the materials submitted to hydrothermal ageing procedures. For SEC analysis, the material was dissolved in tetrahydrofuran (THF) at 40 °C with a concentration of 1 mg·mL^−1^. This step was followed by the filtration of the prepared material solution using a syringe and an Acrodisk filter (pore size of 0.45 μm). The used SEC instrument was an Agilent Technologies series 1200 (Santa Clara, CA, USA) working with a differential refractive index detection and a linear column (PLgel 5 mm Mixed-D, 200 Da < $M_{w}$ < 400 kDa). Each measurement was repeated at least three times.

#### 2.3.2. Proton Nuclear Magnetic Resonance

The changes in the chemical structure of PLA, LM, and My during extrusion procedures were evaluated by proton nuclear magnetic resonance (^1^H NMR). The materials (raw PLA, raw LM, raw My, and injected PLA blend specimens) to be analyzed were dissolved in deuterated chloroform CDCl_3_ (containing 0.03% of trimethylsilane) at a concentration of 50 mg·mL^−1^. ^1^H NMR spectra were recorded using a Bruker Avance-III HD spectrometer (Rheinstetten, Germany) operating at a ^1^H frequency of 600 MHz. The delay between the scans was 10 s.

#### 2.3.3. Micro-computed x-Ray Tomography

The possible presence of plasticizer inclusions in the bulk of PLA blends was visualized by micro-computed x-ray tomography (µCT) [10]. To this end, 1 to 2 mm-thick specimens were cut from the injected specimens and analysed with an EasyTom 160 from RX Solutions (Chavanod, France). The acquisition of radiographic images was conducted during four hours at a tube voltage of 50 kV, current of 40 µA and power of 2W enabling to have a good contrast in the case of polymeric materials. For the imaging, the samples were rotated by 360° with an angular resolution of 0.25°, while an image plate detector was utilized. The 3D volume was reconstructed by means of the software Xact64 (RX Solutions) after performing the inherent treatments such as geometrical corrections, removing the ring artefacts, and increasing contrast. The voxel size of the reconstructed volume was comprised between 2.5 µm and 5.44 µm. Finally, we extracted representative 2D slices from the reconstructed volume by means of Xact64. Such an analysis was also conducted on the fractured tensile specimens of the different PLA materials to identify any deformation heterogeneities responsible for the fracture.

#### 2.3.4. Atomic Force Microscope

The microstructure of the injected PLA blend samples was characterized by means of an atomic force microscope (AFM) Asylum MFP-3D Infinity (Santa Clara, CA, USA) in bimodal AM-FM mode. Only topographical images were reported in this paper. For these measurements, the sample surface was polished with a cryo-ultramicrotome Leica EM UC6/UF6 (Wien, Austria) at -50 °C by using first a glass knife for rough polishing and then a diamond blade (Diatome Cryo 35°) for fine polishing. Topography was recorded by keeping the amplitude of the cantilever first resonance frequency around 60 nm from a 100 nm free amplitude to allow the contact with the sample to be in a repulsive mode. Images of 512 px × 512 px were recorded at a line speed of 1 Hz.

#### 2.3.5. Differential Scanning Calorimetry

The thermal properties of PLA blends were evaluated by differential scanning calorimetry (DSC) using a Netzsch DSC 204 F1 instrument (Selb, Germany) with a nitrogen gas flow. DSC testing was done on the injected PLA materials. To this end, samples with a mass comprised between 3 mg and 6 mg were heated from −100 °C to 180 °C with a heating rate of 10 °C·min^−1^, cooled down from 180 °C to −100 °C at −10 °C·min^−1^, and subjected to a second heating stage from −100 °C to 180 °C at 10 °C·min^−1^. Attention was focused on the measurement of the glass transition temperature of PLA ($T_{g}$), the cold-crystallization temperature of PLA ($T_{\mathrm{cc}}$), and the melting temperature of PLA ($T_{m}$). Last, the crystallinity $X_{c}$ of PLA was calculated from the difference between its melting ($\Delta H_{m}$) and cold-crystallization ($\Delta H_{\mathrm{cc}}$) enthalpies, based on the equation:

(7) $$X_{c}=\;\frac{{\Delta H}_{m}\;-{\Delta H}_{\mathrm{cc}}}{f_{\mathrm{PLA}}\times {\Delta H}_{m}^{o}}$$

where $\Delta H_{m}^{o}$ was the enthalpy of a 100% crystalline PLA polymer taken to be equal to 93 J·g^−1^, and $f_{\mathrm{PLA}}$ was the effective weight fraction of PLA, which was 0.79 for the reactive compositions or 0.80 for the non-reactive compositions. Each measurement was repeated three times.

#### 2.3.6. Mechanical Testing

The tensile behavior of the PLA materials was measured at room temperature (22 °C) and at a strain rate of 6 mm·min^−1^ with a universal electromechanical testing machine Instron 5967 (Norwood, MA, USA). Based on the evolution of the engineering axial stress $\sigma_{\mathrm{eng}}$ as a function of the engineering axial strain $\varepsilon_{\mathrm{eng}}$, the tensile modulus E, the yield stress $\sigma_{y}$, the ultimate strain $\varepsilon_{u}$, and the ultimate stress $\sigma_{u}$ of the materials could be determined. To this end, the data evaluation software Instron was used. Concerning impact testing, the injected PLA material samples were first notched with an Instron Ceast Motorized Notchvis machine (Norwood, MA, USA) using a notch radius of 25 mm and a notch angle of 45 °. Then, the samples were impacted by means of a pendulum Instron Ceast 9050 (Norwood, MA, USA) at room temperature (22 °C) based on ISO 180 standard. The IZOD notch impact strength $a_{\mathrm{iN}}$ was assessed with the pendulum integrated software. Each testing was repeated three times.

### 2.4. Ageing

Hydrothermal ageing was conducted to investigate PLA blends stability over short periods [26]. For this procedure, PLA material flakes (with a unit mass of around 1 mg) were first dried at 60 °C in a vacuum oven until a constant mass was attained. Then, the dried flakes were immersed into distilled water (volume of 20 mL) and submitted to a constant temperature of 70 °C (above the glass transition temperature and below the cold-crystallization temperature of PLA) in the Thermo scientific Heraeus oven. The samples were periodically weighed with a precision balance (precision of 0.1 mg) operated at room temperature. To this end, the samples were removed from the water and were wiped with a clean and dry cloth to remove the surface water. The percentage of water uptake at any time t, $M_{t}$, was calculated by:

(8) $$M_{t}\;=\;\frac{m_{t}\;-\;m_{o}}{m_{o}}\;\times \;100$$

where $m_{o}$ and $m_{t}$ denoted the mass of the dry material (the initial mass prior ageing) and the mass of materials after exposure to water absorption, respectively. This measurement was performed from five flakes per ageing step. Aged samples were also characterized by SEC and DSC based on the methods described in the characterization section. It is to be noted that during this ageing procedure, the pH of the distilled water was not controlled, which was a limitation of our hydrothermal procedures.

## 3. Results and Discussion

### 3.1. Processability

**Figure 1:**
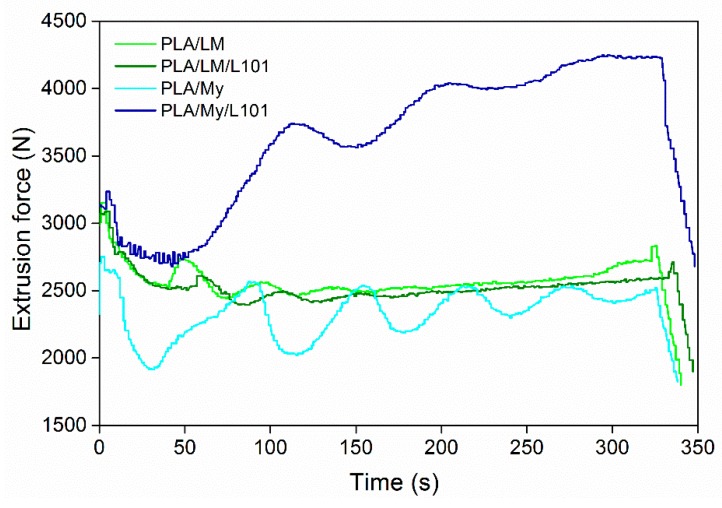
Typical evolution of the extrusion force as a function of the extrusion time, time origin corresponding to the injection of liquid (plasticizer with or without the radical initiator) to polylactide (PLA) already present in the micro-compounder.

As starting point, it is important to verify the processability of the PLA-based materials by monitoring the extrusion force or torque as a function of the extrusion time, by quantifying degradation from molecular weight measurements after each processing step, and by observing the physical aspect of the final shaped materials. The evolution of the extrusion force with time can provide rough information about the material viscosity. In particular, if the force drastically increases during the extrusion, chemical reactions of the PLA matrix, and/or of the plasticizer, and/or between the PLA matrix and the plasticizer can be expected. In the opposite case, an important decrease of the force may be a sign of thermal degradation of PLA [27] and/or the plasticizer. Extrusion force–time curves were provided in Figure 1 for PLA/LM 80/20, PLA/LM/L101 79/20/1, PLA/My 80/20, and PLA/My/L101 79/20/1. Just after the injection of the liquid plasticizer or plasticizer/initiator mixture into the extruder, the recorded extrusion force decreased significantly, and then, exhibited some oscillations for all the materials. These oscillations tended to disappear with time, so that the force reached a final value slightly lower than the initial one prior liquid injection in the case of PLA/LM 80/20, PLA/LM/L101 79/20/1, PLA/My 80/20. In the case of PLA/My/L101, the extrusion force significantly increased after the initial step of force decrease.

Our results demonstrate that extrusion mainly involves a homogenization of PLA/LM, PLA/LM/L101, and PLA/My, without significant reaction or degradation modifying the viscosity. Nevertheless, in the case of PLA/My/L101, the extrusion force increase reflects some marked reactions inducing an increase of viscosity. As mentioned in the introduction section, My is highly reactive and can spontaneously polymerize by thermally activated radical polymerization [17,18]. It is also shown in the literature that My polymerization in the presence of a radical initiator yields poly(myrcene) (pMy) with different possible structures [28]. Such a reaction may be dominant in the case of PLA/My/L101. The nature of the reactions occurring during reactive extrusion will be discussed in the next sections.

**Figure 2:**
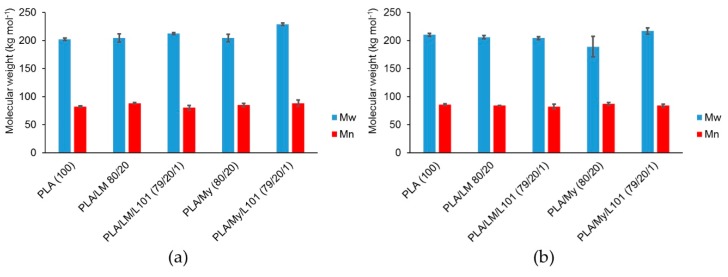
Molecular weights $M_{w}$ and $M_{n}$ of polylactide (PLA)-based materials determined by size exclusion chromatography (SEC) after extrusion (**a**), and after extrusion and injection molding (**b**) (with standard deviations).

To further characterize the possible lack of degradation or the possible reactions occurring during the extrusion step, SEC testing was conducted to measure the molecular weight of PLA-based materials (Figure 2a). Such measurements were also done after the injection step (Figure 2b). In the case of PLA, $M_{w}$ was found to be 202 kg·mol^−1^ after extrusion (Figure 2a) and 210 kg·mol^−1^ after the injection step (Figure 2b). Knowing that the initial molecular weight of PLA 4043D pellets was 199 kg·mol^−1^ [29], the inherent precision of SEC measurements being a few percent, and the standard deviation for each material being around 3%, no significant degradation occurred with the current processing conditions. Moreover, no influence of the addition of LM or My on PLA molecular weight was noted after the extrusion step, confirming that extrusion mainly involved homogenization of the blends without degradation (Figure 1). In the case of the reactive extrusion in the presence of L101, a weak but significant increase of $M_{w}$ was detected for PLA/LM/L101 ($M_{w}$ = 212 kg·mol^−1^) and PLA/My/L101 ($M_{w}$ = 229 kg·mol^−1^) compared to PLA/LM ($M_{w}$ = 204 kg·mol^−1^) and PLA/My ($M_{w}$ = 204 kg·mol^−1^), respectively.

According to previous works [30], an increase of PLA molecular weight during reactive extrusion can be attributed to the free radical initiated branching of PLA. It can also be hypothesized that this potential increase of PLA molecular weight may be attributed to the grafting of the plasticizers onto PLA backbone. Nevertheless, based on the weak increase of $M_{w}$, such reactions may be quite limited.

**Figure 3:**
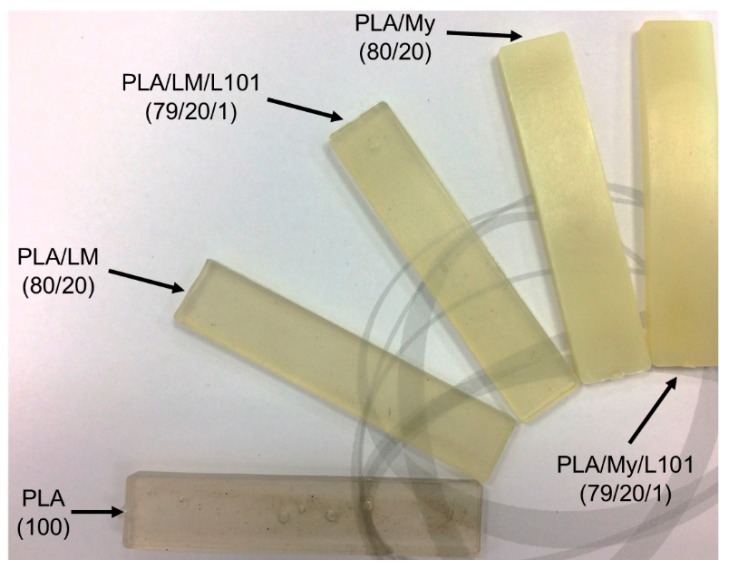
Photograph of as-processed polylactide (PLA)-based materials after injection molding showing that PLA, PLA/limonene (PLA/LM), and PLA/LM/luperox 101 (PLA/LM/L101) were transparent, while PLA/myrcene (PLA/My) and PLA/My/luperox 101 (PLA/My/L101) were opaque (specimen width 11 mm).

A picture of the injected specimens was provided in Figure 3. All the materials exhibited a homogeneous shape and aspect indicating good processing conditions and mixing. It can be seen that PLA/LM, and PLA/LM/L101 were quite transparent and slightly green, while PLA/My and PLA/My/L101 appeared opaque and slightly yellow. 

The transparency of PLA/LM and PLA/LM/L101 can be explained by a low crystallinity level and no or very small inclusions of plasticizer that do not scatter light. The opposite case can also be supposed for PLA/My and PLA/My/L101. The microstructure of the plasticized PLAs will be studied in the next section to elucidate these aspects. It can however be concluded that PLA/My or PLA/My/L101 may be suitable for opaque packaging applications, while PLA/LM or PLA/LM/L101 may be suitable for transparent packaging applications. 

### 3.2. Chemical Structure and Microstructure 

To assess the chemical structure of PLA-based materials after reactive extrusion, it was suitable to combine direct methods such as Fourier transform infrared spectroscopy (FTIR) and ^1^H NMR, and indirect methods such as swelling testing and microstructural investigations to gather a maximum of information. 

PLA and terpenes being soluble in chloroform, some swelling testing of the plasticized PLA was done with this solvent at room temperature to qualitatively characterize the presence of a gel, and hence, of a crosslinked network. In the case of PLA, PLA/LM, and PLA/LM/L101, the dissolution of the material was total in chloroform, while in the case of PLA/My and PLA/My/L101, dissolution in chloroform induced the formation of a turbid solution.

Therefore, a crosslinked molecular network in PLA/My and PLA/My/L101 is expected, resulting in a very low gel fraction, which can be due to crosslink formation in the PLA matrix or crosslink formation in the pMy inclusions [28]. Concerning PLA/LM/L101, crosslinks in the PLA matrix or in the LM phase may not be sufficiently present to induce a gel formation in chloroform.

FTIR measurements in attenuated total reflectance (ATR) mode from 400 cm^−1^ to 4000 cm^−1^ were conducted to identify any potential change of chemical bonds of PLA matrix after reactive extrusion with LM or My (results not shown here). However, no significant change was noticed compared to neat PLA. Concerning ^1^H NMR investigation (curves provided in the Supplementary Materials), in the case of the PLA-myrcene system, the spectra of the extruded samples with and without L101 showed no evidence of other signals than those arising from the starting materials or impurities contained therein (Figure S1). One of these impurities was pMy [31], and we can thus not conclude whether any polymerization of myrcene is taking place during the extrusion process. Comparison of the ^1^H NMR spectra of the PLA-limonene system with or without the accelerator L101 with those of the starting materials did not reveal any crosslinking between the two ingredients (Figure S2).

Based on FTIR and ^1^H NMR results, direct methods enabling to identify the chemical structure of the PLA materials do not allow to extract information about the change in PLA and plasticizer structure during the extrusion. One explanation may be the total absence or very diluted structural changes, so that they cannot be detected.

**Figure 4:**
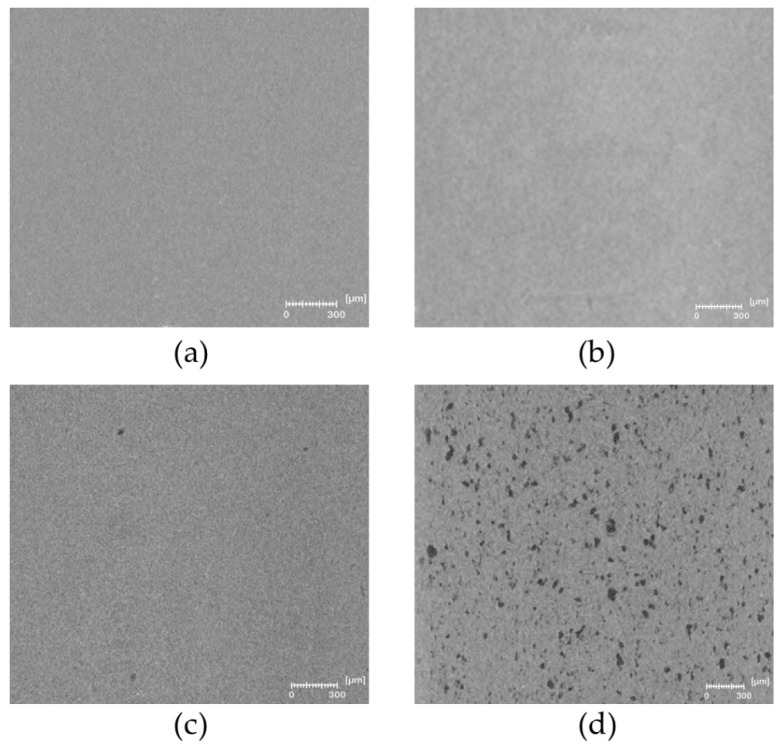
Representative 2D slices extracted from the reconstructed volume by micro-computed x-ray tomography (µCT) of (**a**) polylactide/limonene (PLA/LM), (**b**) PLA/LM/luperox 101 (PLA/LM/L101), (**c**) PLA/myrcene (PLA/My), and (**d**) PLA/My/luperox 101 (PLA/My/L101).

The microstructure of PLA and plasticized PLA materials was observed by µCT testing (Figure 4). In particular, the density variation of the materials was visualized in 3D. In the case of PLA/LM and PLA/LM/L101, no contrast was noted on the extracted 2D images proving that these plasticized PLA materials have a homogeneous microstructure at the µCT resolution (Figure 4a,b). On the contrary, in the case of PLA/My (Figure 4c), some black spots with a maximum size of 60 µm were observed. The number and size of these black spots significantly increased when adding L101 during the extrusion (Figure 4d). In particular, the largest black spots reached a size of 200 µm. Obviously, these black spots had a lower density compared to the matrix and were considered as plasticizer inclusions.

As predicted by the Hansen Solubility Parameters (Table 1), LM was more soluble in PLA compared to My, explaining the presence of micrometric inclusions in PLA plasticized with My that were not present in PLA plasticized with LM at the µCT resolution. Furthermore, the presence of L101 seems to promote the polymerization of myrcene.

**Figure 5:**
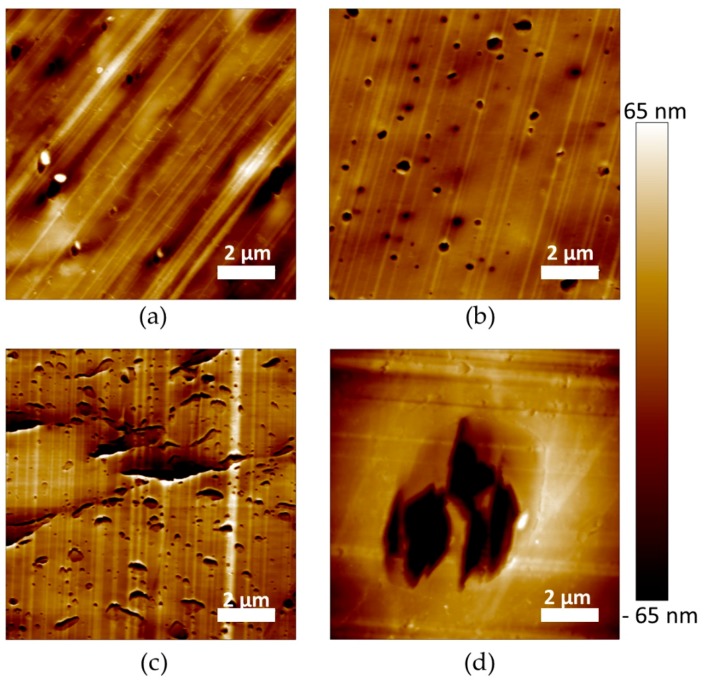
Atomic force microscopy (AFM) imaging in topography mode of (**a**) polylactide/limonene (PLA/LM), (**b**) PLA/LM/luperox 101 (PLA/LM/L101), (**c**) PLA/myrcene (PLA/My), and (**d**) PLA/My/luperox 101 (PLA/My/L101).

The microstructure of the materials was also characterized at a lower scale by AFM imaging (Figure 5). The images were recorded with the topographical contrast mode. All the plasticized PLA materials exhibited inclusions with a heterogeneous size, considered as being the plasticizer phase (Figure 5a–d). Very large inclusions were obtained for PLA/My/L101 confirming µCT observations (Figure 4d and Figure 5d). In some cases, the inclusions were properly cut by the cryo-ultramicrotome polishing procedure showing a homogeneous internal structure (Figure 5c), while in the opposite case the plasticizer inclusions were totally removed by the preparation method conducting to holes (Figure 5b). Intermediate cases were also noted for which the inclusion internal structure was damaged and exhibited some fibrils (Figure 5d).

### 3.3. Thermal and Mechanical Behaviours

**Figure 6:**
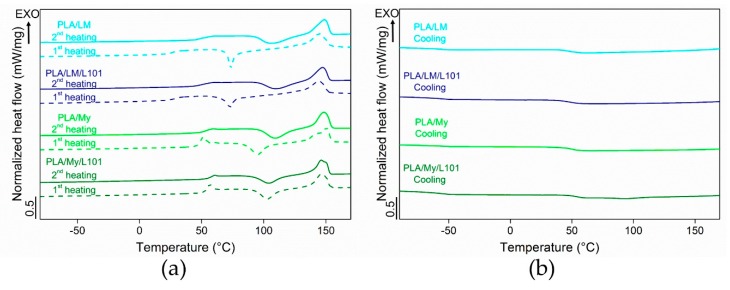
Representative differential scanning calorimetry (DSC) thermograms of polylactide (PLA)-based materials corresponding to the heating stages (**a**) and the cooling stage (**b**).

Some representative DSC thermograms of the materials were represented in Figure 6, while the extracted data was listed in Table 2. In particular, the thermal properties of PLA-based materials were measured during the first heating stage, the subsequent cooling stage, and the second heating stage. During the cooling stage of all the PLA-based materials, only the glass transition was noted (no crystallization peak). In the case of PLA, the measured $T_{g}$ was 54.4 °C ± 0.4 °C. All the plasticized PLAs exhibited a lower $T_{g}$ compared to neat PLA. In the case of PLA plasticized with LM, a $T_{g}$ of 46.5 ± 1.3 °C was obtained for PLA/LM and a $T_{g}$ of 48.1 ± 1.1 °C was obtained for PLA/LM/L101. In the case of PLA plasticized with My, a of 50.5 ± 0.5 °C was obtained for PLA/My and a $T_{g}$ of 52.6 ± 0.5 °C was obtained for PLA/My/L101. Therefore, in the case of PLA/LM, the glass transition temperature is quite unaffected when adding L101, while in the case of PLA/My, $T_{g}$ was few degrees higher in the case of the reactive blending in comparison with the non-reactive one.

Our results indicate that LM and My, with or without L101, enable to plasticize PLA according to the obtained glass transition temperature, lower for plasticized PLAs compared to neat PLA. The more marked decrease of $T_{g}$ results from LM, as this plasticizer presents a better miscibility with PLA compared to My (Table 1). Extrusion in the presence of L101 conducts to an increase of $T_{g}$ for My that can be due to the enhanced My polymerization (Figure 4c,d). Indeed, the dispersion of My within PLA, and hence the availability of My for increasing the PLA chains’ free volume is supposed to be reduced by the reactive extrusion. In the case of LM, the addition of L101 during the extrusion seems to have no effect on LM polymerization, confirming extrusion force monitoring (Figure 1), and microstructure observations (Figure 4a,b and Figure 5a,b).

During the heating stages, differences between the two families of plasticized PLA were more marked. PLA/LM and PLA/LM/L101 exhibited a strong decrease of $T_{g}$ to a value below 30 °C in the first heating stage. Interestingly, the $T_{g}$ was in the cooling stage observed at over 40 °C and finally in the second heating stage it was observed at around 50 °C, just 10 °C lower than for PLA having a $T_{g}$ of 60.9 ± 0.1 °C. The same behavior was noted concerning the cold-crystallization temperature $T_{\mathrm{cc}}$. Indeed, a low $T_{\mathrm{cc}}$ was measured at around 70 °C in the first heating stage, while in the second heating stage, the $T_{\mathrm{cc}}$ was higher than 100 °C for both PLA-limonene materials. The melting temperature increased just by a few degrees from the first to the second heating stage for PLA/LM and PLA/LM/L101. It is obvious that the cold-crystallization was characterized by a narrow peak in the first heating stage and by a broad peak in the second heating stage. Overall, the effect of the addition of the radical initiator L101 on the thermal properties of PLA/LM was very low, since all the thermal properties were quite similar for PLA/LM and PLA/LM/L101. This supports the previous interpretation of the extrusion force, where no difference between the non-reactive and the reactive blending was observed in the case of LM (Figure 1), although the crystallinity increased from PLA/LM to PLA/LM/L101. This increase of crystallinity is probably due to a weak branching/crosslinking level of PLA. Indeed, a low concentration of compact grafting/crosslinking regions may act as crystallization precursor, enhancing PLA crystallization [32,33].

In the case of My-plasticized PLA materials, higher glass transition temperatures were observed in the first heating stage compared to the LM-plasticized PLA materials and just an increase by a few degrees was measured in the second heating stage. Furthermore, in the second heating stage, the glass transition temperature of PLA/My equal to 54.3 ± 0.4 °C and of PLA/My/L101 equal to 57.1 ± 0.5 °C were just a few degrees lower than in the case of PLA ($T_{g}$ = 60.9 ± 0.1 °C). It is also to be noted that the glass transition occurring in the first heating stage was accompanied by a small endothermal peak, which disappeared in the second heating stage. This additional peak, also observed in the case of neat PLA, was attributed to the melting of a mesophase or to an ageing phenomenon [34,35]. For PLA/My blend, the cold-crystallization temperature increased by 13 °C from the first to the second heating stage. In the case of PLA/My/L101 material, just a 3 °C increase was noted between the first and the second heating stage. This stabilization of $T_{\mathrm{cc}}$ was the only relevant finding when comparing the non-reactive blend with the reactive blends of PLA with myrcene. During the first heating stage, both PLA/My and PLA/My/L101 exhibited a double melting peak resulting from two families of crystals, which transformed into a single melting peak during the second heating stage. As a result, the heating step (melting) followed by the cooling step (crystallization) may have homogenized the crystals into one unique family. Furthermore, the crystallinity was slightly higher for the non-reactive blend PLA/My compared to the reactive blend PLA/My/L101 during the first heating stage. Although the weak branching of PLA is supposed to fasten PLA crystallization as for PLA/LM/L101, the presence of L101 is also supposed to accelerate My polymerization. The formation of pMy inclusions may decrease the availability of My to ensure a good chain mobility of PLA and may tend to hinder PLA crystallization at a given step of crystallization. So, pMy is limiting PLA crystallization, despite PLA crystallization is initially accelerated by the presence of the compact grafting/crosslinking regions [32,33]. However, during the second heating stage, the opposite result was obtained. It is suggested that My plasticizer in PLA/My has been further polymerized (higher conversion of the monomers) by the first heating stage of DSC measurements, conducting to less available plasticizer for PLA matrix, and hence, less plasticization and chain mobility to attain a high crystallinity. Such a phenomenon is less probable in PLA/My/L101 for which polymerization may reach its limit during the extrusion step.

**Figure 7:**
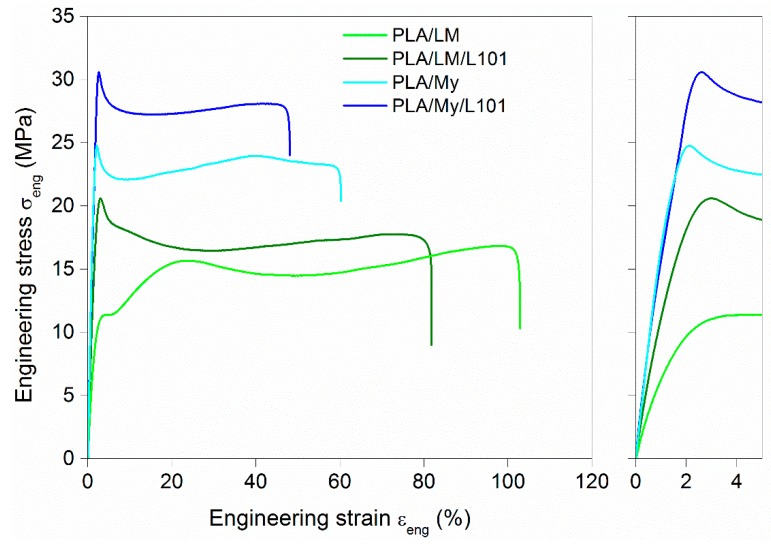
Representative engineering stress vs. engineering strain curves of polylactide (PLA)-based materials obtained in tension at 22 °C and 6 mm·min^−1^ (a zoom-in view of the curves highlighting strain levels below 5% was provided).

It is now important to investigate the effect of adding My and LM into PLA, with or without radical initiator, on the tensile and impact properties of the PLA-based materials. The engineering stress–engineering strain curves of the materials were plotted in Figure 7, while the extracted parameters were listed in Table 3. Compared to neat PLA, the addition of plasticizer, with or without L101, conducted to an important decrease of the elastic modulus E, decrease of the stress at yield $\sigma_{y}$, increase of the ultimate strain $\varepsilon_{u}$, and decrease of ultimate stress $\sigma_{u}$. The intensity of these variations depended on the plasticization method (physical blending vs. reactive blending) and of the type of plasticizer (My vs. LM). The differences between LM and My were quite marked. Indeed, the ultimate strain of PLA/LM was almost the double of that of PLA/My. In the case of LM, the ultimate strain was similar for both plasticization methods ($\varepsilon_{u}$≈ 120%), and in the case of My, the ultimate strain was about one quarter lower for the reactive blending ($\varepsilon_{u}$= 45.0%) compared to the non-reactive blending ($\varepsilon_{u}$ = 62.7%). The tensile modulus for LM-plasticized materials (E = 1.0 GPa for PLA/LM and E = 1.2 GPa for PLA/LM/L101) was lower compared to PLA (E = 2.3 GPa), and E was slightly higher in the case of the use of L101 as shown previously with acryl-PEG [8]. In the case of My-plasticized materials, E was comprised between that of PLA and that of LM-plasticized materials (E = 1.9 GPa and E = 1.7 GPa for PLA/My and PLA/My/L101, respectively). Contrary to what was observed for LM-plasticized PLA, the tensile modulus of the non-reactive PLA/My blend was slightly higher than that of reactive PLA/My/L101 blend. Concerning impact behavior, the addition of LM or My to PLA improved the impact strength $a_{\mathrm{iN}}$ compared to neat PLA for which $a_{\mathrm{iN}}$ was equal to 2.7 ± 0.1 kJ·m^−2^. The highest impact strength, $a_{\mathrm{iN}}$ = 12.5 ± 0.9 kJ·m^−2^, was achieved in the case of PLA/My, while for the other cases the impact strength was comprised between 4.9 ± 0.1 kJ·m^−2^ and 5.8 ± 0.6 kJ·m^−2^.

**Figure 8:**
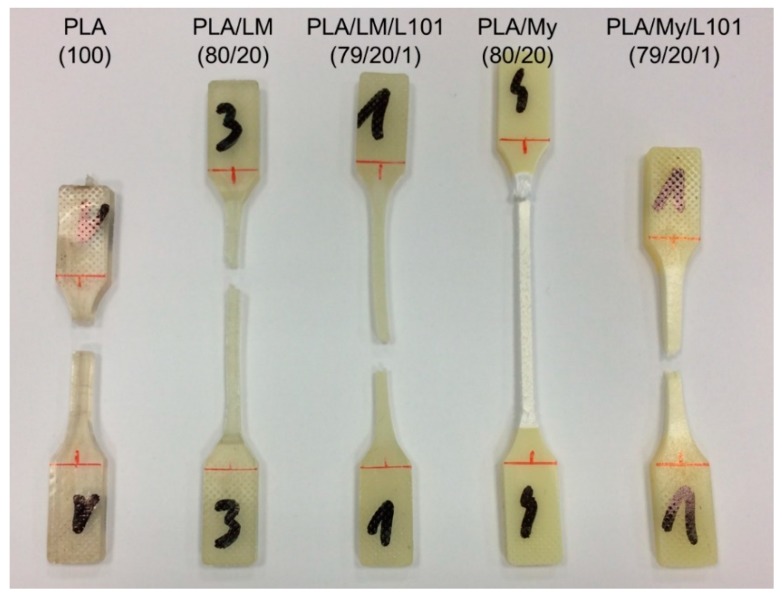
Pictures of drawn tensile specimen until failure of polylactide (PLA)-based materials.

**Figure 9:**
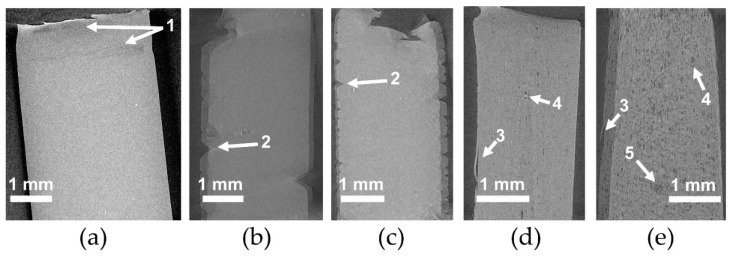
Representative 2D slices extracted from the reconstructed volume by micro-computed x-ray tomography (µCT) of broken tensile specimen of (**a**) polylactide (PLA), (**b**) PLA/limonene (PLA/LM), (**c**) PLA/LM/luperox 101 (PLA/LM/L101), (**d**) PLA/myrcene (PLA/My), and (**e**) PLA/My/luperox 101 (PLA/My/L101). 1: crazing, 2: cracking from the surface, 3: surface delamination, 4: plasticizer-matrix debonding forming cavities, and 5: coalescence of cavities induced by the plasticizer–matrix debonding (tensile direction is vertical).

The observation of the tensile specimen after breakage can also provide information about the influence of the PLA plasticization methodology on the mechanical behavior, and in particular, on the deformation heterogeneities. A picture showing the broken tensile specimen of PLA, PLA/LM, PLA/LM/L101, PLA/My, and PLA/My/L101 was shown in Figure 8. PLA tensile specimen clearly exhibited limited deformation and no particular change of aspect remaining transparent. Concerning the tensile specimen of PLA plasticized with LM, it exhibited important deformation, while the initial transparency and green color were retained. Finally, in the case of PLA plasticized with My, an important whitening of the broken tensile specimen was noted. These specimens were further investigated by µCT to identify more clearly deformation heterogeneities (Figure 9). In the case of neat PLA (Figure 9a), the presence of crazes was noted by µCT confirming previous study [36]. In the case of PLA plasticized with LM, the presence of cracks generated from the surface were noted (Figure 9b,c). In the case of PLA plasticized with My, other deformation heterogeneities were observed, namely surface delamination (Figure 9d,e), plasticizer–matrix debonding forming cavities (Figure 9d,e), and coalescence of these cavities (Figure 9e).

Obviously, the plasticization of PLA with LM or My, with or without radical initiator, has increased PLA ductility and impact strength. Prior to modification, PLA exhibited a brittle tensile behavior characterized by crazing development to withstand the imposed macroscopic strain, since chain mobility was very low [35]. The presence of LM or My seems to significantly increase PLA chain mobility since plastic deformation of PLA is active and crazing was no more observed. Nevertheless, some cracks developing from the tensile specimen surface are noted in PLA/LM (Figure 9b). These deformation heterogeneities may be generated to withstand the imposed strain once the chain orientation mechanisms may not be active anymore. Concerning PLA/My, it exhibits inclusion-matrix debonding forming cavities, as deformation heterogeneities appearing to withstand large strain. It is possible that the propagation and coalescence of such cavities engender the formation of surface debonding as observed in Figure 9d. The formation of such cavities may also explain the whitening phenomenon of this material as shown in Figure 8. The higher ultimate strain of PLA/LM compared to PLA/My can be due to the larger size of My plasticizer inclusions compared to LM inclusions (Figure 4a,c and Figure 5a,c) and higher miscibility of PLA with LM compared to My (Table 1). As a result, LM plasticizer-PLA matrix debonding may not be present in PLA/LM (no whitening phenomenon) or appears at a nanometric scale not captured by µCT and does not scattering light. Cavities forming at the micron scale in PLA/My may be generated at a moderate imposed strain and may propagate very fast conducting to an early failure compared to PLA/LM in which nanometric debonding may slowly propagate and/or surface cracks may not be generated yet. In the case of My, the extrusion in the presence of L101 decreases the ultimate strain compared to the extrusion without L101 as the plasticizer inclusion size increases, enhancing cavitation by debonding and reducing ductility (Figure 9d,e). In the case of LM, the presence of L101 does not influence the elongation at break, confirming that no significant effect of LM structure is induced by L101 during the extrusion except some very local grafting/crosslinking. For the two plasticizers, reactive extrusion conducts to an increase of the stress at yield and the ultimate strength. In the case of LM, this is probably engendered by the increased crystallinity compared to the extrusion without L101 (Table 2). In the case of My, crystallinity being almost the same between PLA/My and PLA/My/L101, the increase of strength occurring after reactive extrusion may be linked to the increase of $T_{g}$ (Table 2). Indeed, as stated in the DSC section, the formation of pMy makes less My available for the PLA plasticization.

The increase of PLA chain mobility resulting from the addition of plasticizer is reflected by an increase of impact strength in comparison with neat PLA. Nevertheless, a remarkable result has been obtained in the case of PLA/My that exhibits the highest impact strength among plasticized PLA materials. It is thought that this finding is due to the plasticizer inclusion–PLA matrix debonding acting as an additional dissipation mechanisms to chain rearrangement during the impact. This is true as soon as the cavitation produced at the inclusion-matrix interface does not propagate too fast to neighboring cavities. Such a mechanism implies that inclusions are small and well dispersed. This is not the case for PLA/My/L101 where inclusions are quite big (Figure 4d and Figure 5d), enhancing cavitation propagation and surface delamination conducting to an earlier material failure compared to PLA/My. It is to be noted that the formation of surface delamination in PLA/My can be responsible for the slow decrease of tensile stress from 40% of tensile strain (Figure 7).

### 3.4. Hydrothermal Ageing 

Polyester hydrolysis was followed by the number of chain scissions $n_{t}$ in mol·g^−1^ defined as follows [37]:

(9) $$n_{t}\;=\;\frac{1}{M_{n,t}}-\frac{1}{M_{n,o}}$$

where $M_{n,t}$ represented the number average molecular weight of PLA as a function of ageing time, and $M_{n,o}$ represented the initial number average molecular weight of PLA. $M_{n,t}$ was calculated by SEC in THF at 40 °C based on PS calibration and using the Kuhn–Mark–Houwink equation [38]:

(10) $$M_{n,t}\;=\;0.4055\cdot (M_{n,\mathrm{PS}}{)}^{1.0486}$$

In addition, since hydrolysis of PLA was expected to destroy ester functions, the concentration of the remaining ester functions $e_{t}$ in mol·g^−1^ as a function of time could be calculated according to:

(11) $$e_{t}\;=\;e_{o}\;-\;n_{t}$$

with the initial concentration of esters $e_{o}$ being proportional to the molar mass of PLA repetitive unit ($M_{\mathrm{RU}}$) of 72 g·mol^−1^:

(12) $$e_{o}\;=\;\frac{1}{M_{\mathrm{RU}}}=\;\frac{1}{72}{\;\mathrm{mol}\cdot g}^{-1}$$

Assuming that ester hydrolysis kinetic had a first order for each reactant (ester and water) and degenerated in one of the reactants (the water being constant in concentration), one could write:

(13) $$\ln\left(\frac{e_{o}}{e_{o}{-n}_{t}}\right)\;=\;\ln\left(\frac{1}{{1-72\cdot n}_{t}}\right)\;=\;\mathrm{kt}$$

with k being the kinetic constant of the degenerated order in h^−1^ (considering hour as unit of time) such as:

(14) $$k\;=\;K\cdot e_{o}\cdot \left[H_{2}O\right]=\;K\;\cdot \frac{1}{72}\cdot \frac{1000}{18}$$

In Equation (14), K corresponded to the hydrolysis kinetic constant of the second order in L·mol^−1^·h^−1^. 

**Figure 10:**
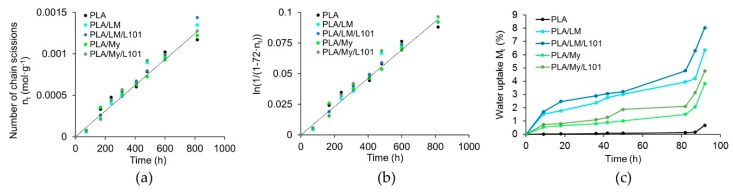
Characterization of polylactide (PLA)-based materials hydrothermal ageing in water at 70 °C. (**a**) Evolution of PLA number of chain scissions $n_{t}$ as a function of time, (**b**) hydrolysis kinetic linear regression and (**c**) evolution of the sample water uptake $M_{t}$ as a function of time determined by weighing.

The evolution of the number of chain scission and water uptake of PLA, PLA/LM, PLA/LM/L101, PLA/My, and PLA/My/L101 were represented in Figure 10. All the materials exhibited similar chain scission number evolutions (Figure 10a). It was possible to calculate the k constant of PLA at 70 °C that was equal to 1.18·10^−4^ h^−1^ (coefficient of determination of 0.98), which was in good agreement with the literature (a k value of 1.20·10^−4^ h^−1^ was found in Reference [39] for the same hydrothermal ageing temperature). Besides this, K was estimated to be in the range of 1.53·10^−4^ L·mol^−1^·h^−1^ (Figure 10b). At the early stage of the ageing procedure, the water uptake $M_{t}$ of the different blends deferred. Indeed, $M_{t}$ increased in the following material order: PLA < PLA/My ≈ PLA/My/L101 < PLA/LM ≈ PLA/LM/L101 (Figure 10c). After 3 days of ageing, DSC measurements (based on the first heating stage) revealed that the glass transition temperature of PLA decreased by 10%, while that of all the plasticized PLAs increased (+27% for PLA/LM, + 22% for PLA/LM/L101, +8% for PLA/My, and + 6% for PLA/LM/L101). At the same time, the crystallinity of all the materials reached values comprised between 51 wt% and 61 wt%, excepted for PLA/My/L101 that exhibited a crystallinity of 36 wt%. Note that an important increase in water uptake was observed after 80 h of ageing for all the materials. Last, during the ageing, the water of all the plasticized PLA blends was slightly colored, while that of PLA remained colorless.

Obviously, PLA degradation kinetic is not influenced during the hydrothermal ageing by the plasticization as shown by the chain scission number evolutions. This finding suggests that the initial PLA structure is not drastically modified by the presence of L101. Indeed, if PLA would have been significantly branched by the free radical initiator, its stability against ageing would have increased [30]. In the case of PLA, the decrease of the glass transition temperature and the increase of crystallinity after 3 days of ageing are linked to the chain scission mechanisms providing a higher chain mobility. At the same time, the plasticization markedly influences the water uptake of PLA materials. LM brings more water uptake than My in plasticized PLAs, which can be directly connected to the lower $T_{g}$ of PLA/LM blends compared to PLA/My blends (Table 2). In addition, the increase of $T_{g}$ and the slight coloration of the water in the case of plasticized PLA materials indicate the release of the two plasticizers during the hydrothermal ageing, explaining also the water uptake. The higher increase of $T_{g}$ in the case of PLA/LM blends compared to PLA/My blends demonstrate that LM is probably more released than My during the ageing. This can be explained by the polymerization of My during the reactive extrusion providing a higher stability compared to LM. Note that the increase of $T_{g}$ observed after the hydrothermal ageing of plasticized PLA materials can be related to the increase of $T_{g}$ when comparing the first and the second DSC heating stage of the same materials (Table 2), both cases being due to the release of the plasticizer. The use of L101 has no or a limited effect on the material water uptake during the hydrothermal ageing. Finally, the authors cannot account for the increase of hydrolysis observed after 80 h of ageing, though they believe that this trend can be connected to the increase of pH in the non-buffered media.

## 4. Conclusions

In this work, we developed and characterized new formulations of plasticized PLA with limonene (LM) and myrcene (My) as bio-based plasticizers, by conventional and reactive extrusion. For reactive extrusion, the organic peroxide 2,5-bis(*tert*-butylperoxy)-2,5-dimethylhexane (L101) was used as free radical initiator. The tested compositions were PLA (100), PLA/LM (80/20), PLA/LM/L101 (79/20/1), PLA/My (80/20), and PLA/My/L101 (79/20/1). All the materials did not exhibit degradation during the extrusion step and the subsequent injection step, demonstrating a good processability. PLA plasticized with LM was transparent, and hence, was suitable for transparent packaging applications, while PLA plasticized with My appeared opaque, and hence, was suitable for opaque packaging applications. During the reactive extrusion, three main reactions were possible: (i) free radical initiated branching and crosslinking of PLA, (ii) grafting/crosslinking of the plasticizers onto PLA backbone, and (iii) polymerization of the plasticizer. We addressed these three hypotheses by a multiscale and multidisciplinary investigation of plasticized PLA materials.

It is probable that PLA exhibits a free radical initiated branching and crosslinking during the reactive extrusion. Indeed, (i) SEC measurements show a weak increase of PLA molecular weight between the non-reactive plasticization and the reactive plasticization with LM and My, (ii) PLA/LM/L101 has a higher crystallinity compared to PLA/LM probably induced by compact grafting/crosslinking regions acting as crystallization precursor, and (iii) tensile yield stress and tensile ultimate stress increase from PLA/LM to PLA/LM/L101 as a direct effect of crystallinity increase.

Concerning the possible grafting/crosslinking of the plasticizers onto the PLA backbone, no data enables to prove this reaction in the case of LM and My. The reason may be the total absence or the rare occurrence of this reaction, so that it could not be detected by ^1^H NMR and FTIR. This is also supported by (i) similar thermal properties of PLA/LM and PLA/LM/L101 based on DSC measurements, (ii) no increase of the extrusion force during the extrusion of PLA/LM/L101, (iii) no-gel formation during swelling testing in chloroform for PLA/LM/L101 (these three observations also enable to conclude that LM does not exhibit any polymerization during the reactive extrusion), and (iv) water uptake during the hydrothermal ageing did not vary when comparing non-reactive with reactive blends.

Finally, only My exhibits polymerization during the extrusion with or without L101, as indicated by (i) the drastic increase of the extrusion force in the case of PLA/My/L101 after the injection of the liquid, (ii) the formation of a turbid solution during swelling testing of PLA/My and PLA/My/L101 in chloroform, and (iii) the important increase of My inclusion size from PLA/My to PLA/My/L101 based on µCT testing. The polymerization of My provides a higher stability compared to LM during the hydrothermal ageing of plasticized PLA.

If the addition of the free radical initiator to PLA/My blend increases its tensile strength (yield stress and ultimate stress), it is in general detrimental for the ultimate strain and the impact strength that drop and for the glass transition temperature that increases. This is mainly due to the important polymerization of My yielding very large inclusions that enhance cavitation, and hence, conduct to an early failure of the material. In the case of PLA/LM blend, the addition of L101 induces an increase of its crystallinity, while in general the other properties are not significantly affected. It would be of interest to investigate the biodegradation and/or mechanical recycling of the non-reactive blend PLA/My and of the reactive blend PLA/LM/L101 as potential end-of-life scenarios of those two promising plasticized PLA materials.

## Figures and Tables

**Table 1:** Calculated Hansen Solubility Parameters of polylactide (PLA), limonene (LM), myrcene (My), and poly(ethylene) glycol (PEG) by means of the software HSPiP.

Substance	$\delta_{d}\;(\mathrm{MJ}\cdot m^{-3}{)}^{0.5}$	$\delta_{p}\;(\mathrm{MJ}\cdot m^{-3}{)}^{0.5}\;$	$\delta_{h}\;(\mathrm{MJ}\cdot m^{-3}{)}^{0.5}$	$R_{a}\;(\mathrm{MJ}\cdot m^{-3}{)}^{0.5}$	RED
PLA	17.7	8.7	10	-	-
LM	16.67	1.92	3.2	9.97	0.93
My	16.07	1.86	2.8	10.59	0.99
PEG	17.2	9	7.3	3.08	0.29

**Table 2:** Differential scanning calorimetry (DSC) parameters of polylactide (PLA)-based materials, stage n°1 indicated the first heating, stage n°2 indicated the cooling, and stage n°3 indicated the second heating.

Material	Stage	$T_{g}\;({}^{\circ}C)$	$T_{\mathrm{cc}}\;({}^{\circ}C)$	$T_{m}\;({}^{\circ}C)$	$\Delta H_{\mathrm{cc}}\;(J\cdot g^{-1})$	$\Delta H_{m}\;(J\cdot g^{-1})$	$X_{c}\;(\mathrm{wt}\%)$
PLA (100)	1	60.8 ± 0.6	126.9 ± 0.0	155.4 ± 0.5	4.7 ± 0.1	8.3 ± 1.4	3.9 ± 1.4
	2	54.4 ± 0.4	-	-	-	-	-
	3	60.9 ± 0.1	127.9 ± 0.1	154.5 ± 0.1	2.1 ± 0.8	2.8 ± 0.5	0.8 ± 0.3
PLA/LM (80/20)	1	27.1 ± 0.2	70.5 ± 4.6	144.8 ± 0.5	12.5 ± 5.5	22.0 ± 1.1	10.2 ± 4.8
	2	46.5 ± 1.3					
	3	50.6 ± 0.3	105.1 ± 0.6	148.9 ± 0.1	16.5 ± 0.5	19.9 ± 0.5	3.6 ± 0.1
PLA/LM/L101 (79/20/1)	1	27.5 ± 0.8	71.9 ± 1.2	143.4 ± 1.1	7.3 ± 2.2	21.3 ± 0.3	15.0 ± 2.0
	2	48.1 ± 1.1					
	3	50.7 ± 0.6	109.2 ± 0.3	147.2 ± 0.4	11.8 ± 1.4	18.2 ± 0.6	6.9 ± 0.9
PLA/My (80/20)	1	49.9 ± 1.7	97.1 ± 3.1	142.4 ± 1.8; 150.8 ± 0.4	13.3 ± 1.6	21.1 ± 1.0	8.4 ± 0.7
	2	50.5 ± 0.5					
	3	54.3 ± 0.4	110.5 ± 2.3	148.4 ± 0.3	18.1 ± 0.8	21.5 ± 1.1	3.6 ± 0.2
PLA/My/L101 (79/20/1)	1	54.3 ± 0.4	102.0 ± 0.7	145.2 ± 0.2; 150.5 ± 0.3	11.4 ± 0.5	17.5 ± 1.5	6.5 ± 2.3
	2	52.6 ± 0.5					
	3	57.1 ± 0.5	105.8 ± 2.3	147.9 ± 0.6	13.1 ± 1.8	18.0 ± 0.4	5.2 ± 1.5

**Table 3:** Tensile and impact properties of polylactide (PLA)-based materials.

Materials	Tensile Modulus	Stress at Yield	Ultimate Strain	Ultimate Stress	Impact Strength
	E (GPa)	$\sigma_{y}$ (MPa)	$\varepsilon_{u}$ (%)	$\sigma_{u}$ (MPa)	$a_{\mathrm{iN}}$ (kJ·m^−2^)
PLA (100)	2.3 ± 0.3	70.2 ± 1.5	7.4 ± 1.0	60.6 ± 31.1	2.7 ± 0.1
PLA/LM (80/20)	1.0 ± 0.2	12.6 ± 2.8	117.5 ± 21.5	15.8 ± 2.6	5.5 ± 0.5
PLA/LM/L101 (79/20/1)	1.2 ± 0.2	20.1 ± 1.6	120.2 ± 23.5	17.2 ± 1.9	5.8 ± 0.6
PLA/My (80/20)	1.9 ± 0.1	25.8 ± 1.3	62.7 ± 13.2	18.7 ± 0.8	12.5 ± 0.9
PLA/My/L101 (79/20/1)	1.7 ± 0.2	30.2 ± 0.8	45.0 ± 8.4	24.8 ± 0.9	4.9 ± 0.1

## Supplementary Materials

The following are available online at https://www.mdpi.com/2073-4360/11/8/1363/s1.
