# Supplementary material for: Plasticization of Polylactide with Myrcene and Limonene as Bio-Based Plasticizers: Conventional vs. Reactive Extrusion
Source: Polymers (Basel). 2019 Aug 18;11(8):1363. doi: 10.3390/polym11081363 (PMC6723983; doi:10.3390/polym11081363)
Supplement: Supplementary file 1 [file polymers-11-01363-s001.pdf]

Plasticization of polylactide with myrcene and limonene as bio-based plasticizers: structure and stability

Berit Brüster [1], Yann-Olivier Adjoua [1], Reiner Dieden [1], Patrick Grysan [1], Carlos Eloy Federico [1], Vincent Berthé [1] and Frédéric Addiego [1,*]

[1] Luxembourg Institute of Science and Technology (LIST), Department Materials Research and Technology (MRT), ZAE Robert Steichen, 5 Rue Bommel, L-4940 Hautcharage, Luxembourg; berit_bruester@web.de (B.B.); yolivier@hotmail.com> (Y.A.); reiner.dieden@list.lu (R.D.); carloseloy.federico@list.lu (C.F.); vincent.berthe@list.lu (V.B.)

* Correspondence: frederic.addiego@list.lu; Tel.: +352-275-888-4639 (F.A.)

Keywords: polylactide; plasticization; myrcene; limonene; reactive extrusion; structure; mechanical properties

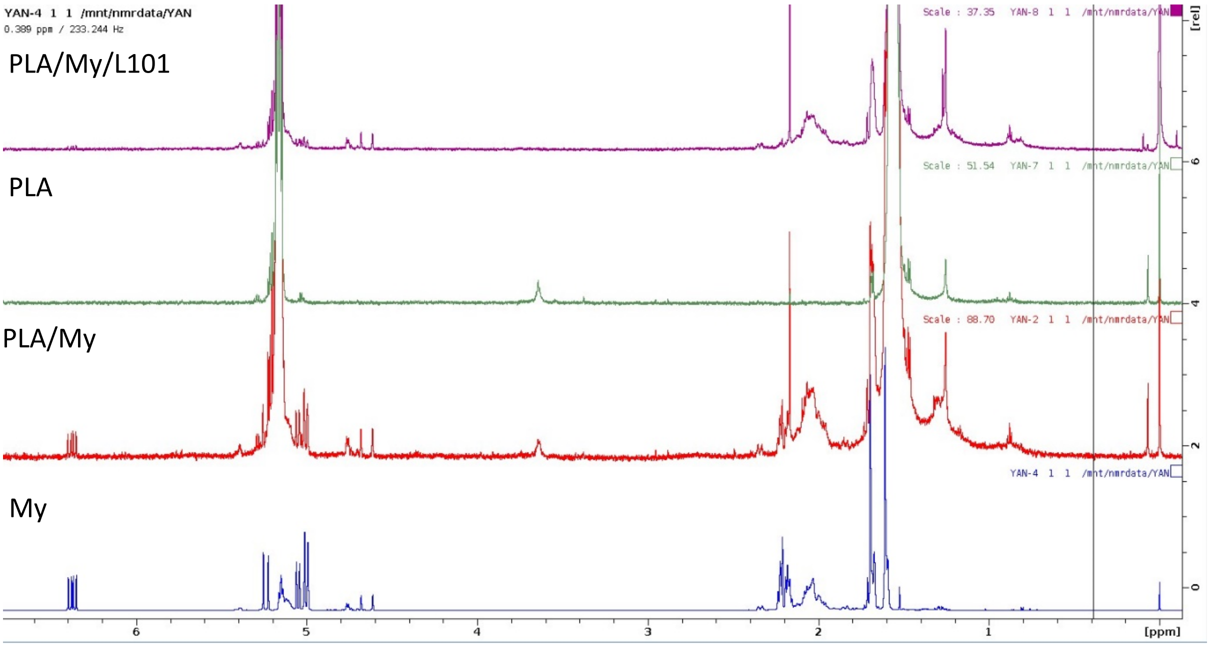

Figure S1: ^{1}H NMR spectra of My, PLA/My, PLA, and PLA/My/L101.

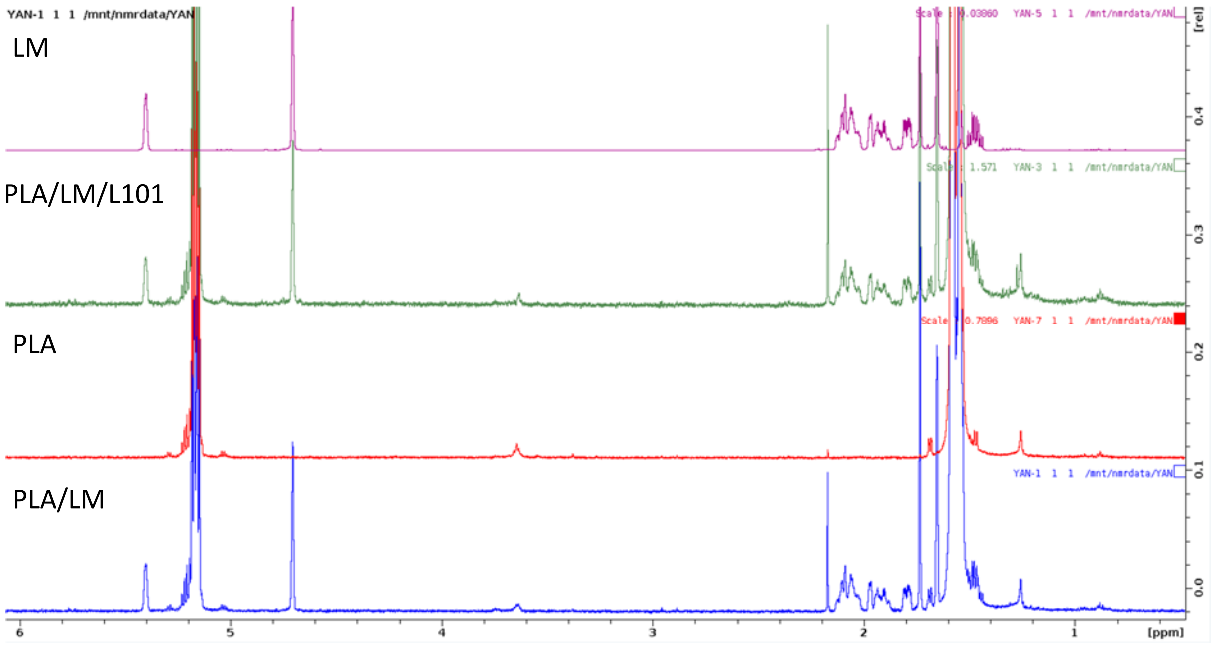

Figure S2: ^{1}H NMR spectra of PLA/LM, PLA, PLA/LM/L101, and LM
